# Short-term impacts and value of a periodic no take zone (NTZ) in a community-managed small-scale lobster fishery, Madagascar

**DOI:** 10.1371/journal.pone.0177858

**Published:** 2017-05-18

**Authors:** Stephen Long

**Affiliations:** 1 SEED Madagascar, Villa Rabemanda, Ambinanikely, Tolagnaro, Madagascar; 2 Department of Geography, University College London, London, United Kingdom; Department of Agriculture and Water Resources, AUSTRALIA

## Abstract

The small-scale lobster fisheries of Madagascar’s impoverished southeast coast account for the majority of national catch and export, making a significant contribution to the regional economy. Data suggests catches have declined, likely due to over-exploitation. In response, the community of Sainte Luce has established a locally managed marine area (LMMA) to manage their lobster fishery, including a 13 km^2^ periodic no take zone (NTZ). Participatory monitoring data were used to assess compliance, identify changes in catch per unit effort (CPUE) associated with the NTZ and consider the short-term value of the NTZ. Compliance is low for measures dictated by national legislation (minimum landing size (MLS), national closed season, prohibition on landing berried females), but may be higher for those designed by the community (NTZ). Upon NTZ opening in July 2015, an estimated 435% increase in catch was observed compared with the mean for the preceding five months, a product of increased effort and significantly higher CPUE. Zero Altered Negative Binomial modelling showed CPUE was significantly higher during the NTZ opening in 2015 and in 2016 when the opening period had been moved. Whilst it is unlikely that tangible ecological benefits have accrued from NTZ operation, there have been important socio-economic effects. Specifically, there was a 33% increase in the price fishers received, a significant effect at the bottom of the value chain. Temporary increases in catch and income acted as a catalyst, engaging neighbouring communities in fishery management, resulting in two additional NTZs. Attention is drawn to the fact that current national legislation may be sub-optimal and should be reviewed. Successful management of the regional fishery will require the state and industry to support communities in adopting community-based management. The NTZ measure considered here may be an effective tool to achieve this.

## Introduction

Small-scale fisheries (SSFs) employ the vast majority of the world’s fishers [1] and account for an increasing proportion of global catch, estimated to be 23% in 2010 [2]. They play an important role in food security, poverty alleviation and nutrition, particularly in developing countries [1, 3, 4]. Compared with industrial fisheries, it has been suggested that SSFs offer a number of advantages in terms of employment, wealth distribution, by-catch, discards and energy consumption [5, 6]. However, factors such as their open-access nature and low barriers to entry make SSFs vulnerable to over-capacity and over-exploitation [7], whilst their complexity, diversity and socio-economic role in communities present management challenges [8]. Developing countries often lack the institutional capacity, political stability or financial resources for effective central governance of SSFs. Thus, there is now increasing focus on bottom-up, decentralised or community-based management approaches [9]. This includes the proliferation of locally managed marine areas (LMMAs), seen over the past few decades in the Indo-Pacific [10, 11], and more recently in Madagascar [12, 13].

Madagascar is one of the poorest countries in the world, ranked 154^th^ of 188 countries in the Human Development Index (HDI), with 87.7% of the population living on less than $1.25 USD day^-1^ [14]. It is one of just eight countries whose real income per capita was less in 2010 than 1960, and the only one of these nations which has not endured conflict [15]. The importance of SSFs in Madagascar has been well documented [13, 16, 17]. It has been conservatively estimated that over 100,000 fishers land 135,000 tonnes annually from fisheries [18]. Despite increasing effort, landings from SSFs may have peaked in Madagascar [16], with many fisheries in decline [15].

Marine resource management in Madagascar has lagged behind terrestrial efforts [13]. As a biodiversity hotspot–noted for its high degree of endemism and rate of habitat loss–the country’s terrestrial ecosystems have gained status as one of the world’s highest conservation priorities [19, 20]. Despite having a coastline of over 5,500 km [12], the country had just three marine protected areas (MPAs) by 2002 [13]. This situation which is rapidly changing, with a 50-fold increase in MPA coverage over the last decade, and increasing efforts to involve communities in marine resource management [21]. As a signatory of the Sydney Promise at the 2014 IUCN World Parks Conference, Madagascar has committed to triple the number of MPAs within the next 5 to 10 years. An initiative to establish community-based octopus (*Octopus cyanea*) fishery management, led to the establishment of Velondriake, Madagascar’s first LMMA [22]. This has been identified as the driver behind the subsequent proliferation of LMMAs in Madagascar [13]. A key component of this was the implementation of short duration (<1 year) time-area closures, or periodic no take zones (NTZs hereafter), pioneered by the Velondriake LMMA [13]. Largely driven by non-governmental organisations (NGOs), the last decade has seen the establishment of a network of over 100 LMMAs. Covering some 12,000 km^2^, these LMMAs are represented nationally by *MItantana HArena and Ranomasina avy eny Ifotony* (MIHARI—Marine resources management at the local level) [12, 23]. Given the infancy and rapid proliferation of LMMAs, community-based fishery management and NTZs in Madagascar, there is a need to assess their efficacy, both ecologically and socio-economically.

Increasing demand for, and the high value of, lobster on international markets has led to the development of commercial lobster fisheries throughout the world [24]. This includes the increase of landings and export through the 1980s from the Fort Dauphin (Tolagnaro) regional lobster fishery. The fishery consists of approximately 40 communities using artisanal fishing practices along the coast of the Anosy and Tandroy regions, between Androka and Manantenina, southeast Madagascar. Lobster exports from Madagascar are of significant economic value, valued at 3.3m USD in 2011, down from a peak in 2007 of $5.1m USD [25]. The Fort Dauphin regional fishery accounts for the majority of the country’s annual lobster catch and export [26] and is a significant contributor to the regional economy, with an estimated 15,000 people directly employed in the lobster industry [27]. Lobster fishing is an important contributor to local economies, playing a crucial role in coastal livelihoods in the southeast [28]. However, limited available data show declines in catch over past decades [29]. This has resulted in attempts to reverse the situation, such as the revision of the national closed season in 2004 [27], and the establishment of a parastatal research body, the Unité de Recherche Langoustière (URL).

The community of Sainte Luce has historically been recognised as one of the key landing sites in the regional lobster fishery [29, 30]. Since 2013, in response to widespread community perception of declining lobster catches and resultant decreases in incomes [31], Sainte Luce has been supported by two NGOs in establishing community-based, sustainable fishery management. This includes the implementation of a NTZ, the first opening of which was in 2014 (August to September, inclusive). The catalyst for establishing the NTZ was a visit by community representatives of Sainte Luce to communities which had established NTZs for octopus on Madagascar’s west coast. Fishers hoped that introducing such a measure would result in increased catches as had been reported for octopus by the communities they visited (E. Ellis, ONG Azafady, pers. comm.). In 2015 the community decided to extend the NTZ opening to three months (July to September, inclusive), whilst in 2016 the community allowed two openings of two months (April to May and August to September inclusive) [32].

This study uses available secondary data and participatory fishery monitoring data (landings, effort and catch composition) from the 2015 and 2016 seasons to consider the community management of this small-scale lobster fishery. The focus is on the NTZ and the change of opening periods between 2015 and 2016, which provided an opportunity to explore the relationship between closures and landings. Specific aims were to: i) assess the short-term impacts of NTZ openings on catch per unit effort (CPUE); ii) consider the ecological and socio-economic value of the NTZ; and iii) where data allow assess compliance with management measures. In addition comparisons are drawn with the established use of NTZs in octopus fisheries on the west coast of Madagascar. Findings will inform on-going management efforts in the local fishery, with applications regionally, as well as to marine resource management across Madagascar and the Western Indian Ocean.

## Methods

### Study site

**Fig 1:**
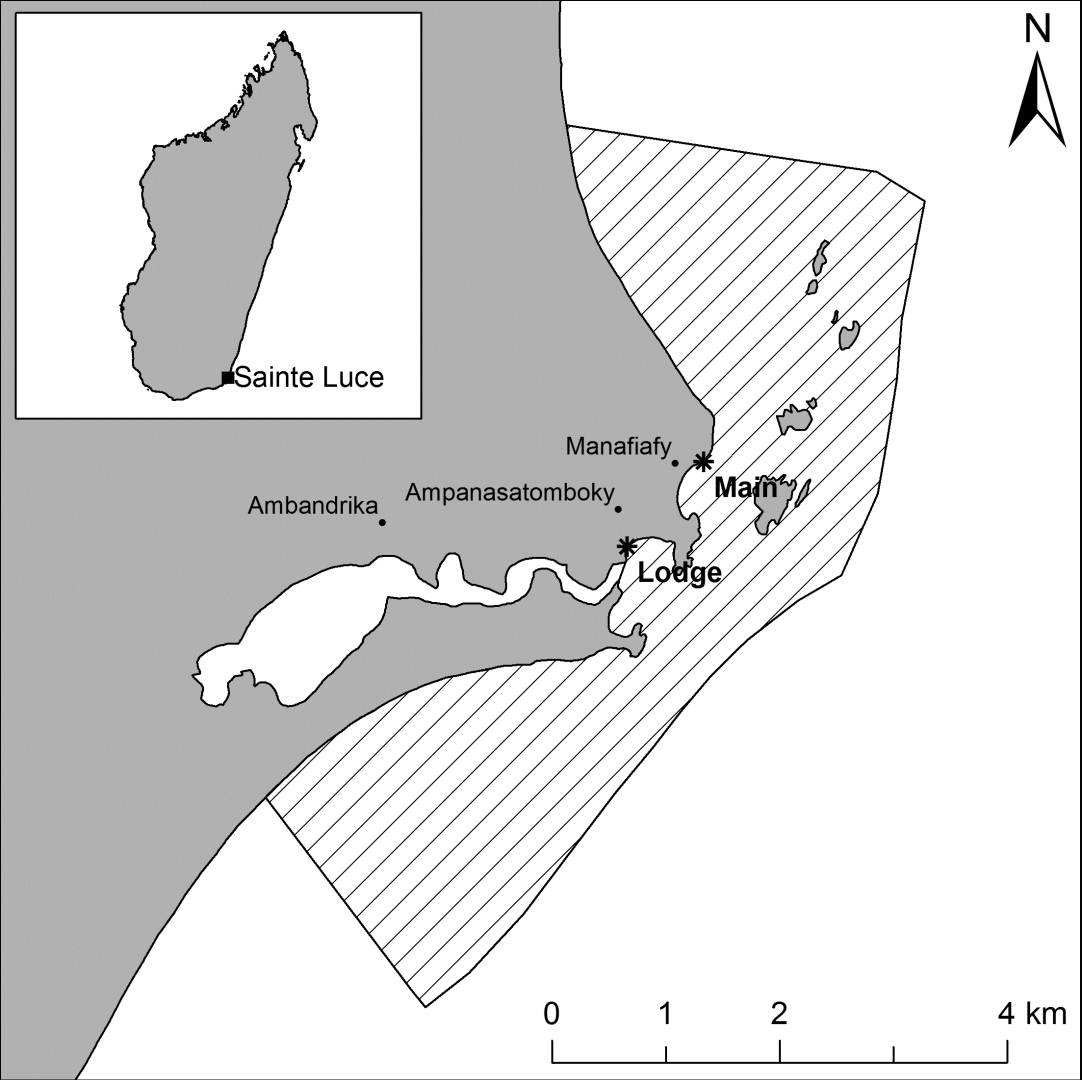
Map of the Sainte Luce lobster fishery. Showing the location of the Sainte Luce lobster fishery, Madagascar. Landing sites are indicated (*) and the no take zone (NTZ) is shown (hashed area).

The community of Sainte Luce is made up of approximately 2,000 people and consists of three hamlets: Ambandrika, Ampanasatomboky and Manafiafy (Fig 1). Fishing is a significant contributor to the community’s economy: around 79% of households identify it as the primary source of income [31], with lobsters being highest value target. Lobsters are caught in traditional pots (approximately 25 x 40 x 70 cm) with a conical entrance, woven from locally available palms or vines [33]. Pots are deployed from traditional wooden *pirogues*, which are constructed from a single hollowed tree trunk and are around 7m in length, referred to as boats hereafter. Fishers, typically between 3 and 5 per boat, leave landing sites at dawn when the wind tends to be lighter and the sea calmer, to check lobster pots deployed overnight. Lobsters are landed at two beaches, Ankatafamirahavavy and Ambatondrahazo, referred to respectively as Main and Lodge throughout, which are situated around a rocky headland and estuary (Fig 1). The lobster fishery operates around patches of rocky reef and a series of small rocky islands, with pots typically set in 3 to 20 m of water. On landing, lobsters are purchased by weight (~$4.50 USD kg^-1^) on the beach by *rabbateurs* employed by *collecteurs* (intermediaries), or purchased directly by *collecteurs*. The *collecteurs* then transport the catch live to Fort Dauphin where they are sold to *opérateurs* (merchants) who process and export the lobster frozen, with a much smaller proportion exported live.

Since 2013, SEED Madagascar and ONG Azafady–British and Madagascan NGOs respectively–have been working in partnership to establish community-based fishery management in the community of Sainte Luce. Accordingly, the fishery is now considered part of MIHARI, Madagascar’s LMMA network [23]. Management responsibility is assumed by a committee of 15 fishers, known as the Riaky Committee [32]. Management measures are stipulated by a *dina* (customary local law) containing 45 articles in addition to *lalana* (national legislation) (Table 1). A *dina* can be described as a code of conduct, developed and enforced by communities [34]. To support improved natural resource governance, *dina* have been incorporated into national law since 1996, meaning they can be formally recognised and enforced by the state following judicial review [35]. The Sainte Luce fishery *dina* was in the process of judicial review at the time of writing, although has *de facto* endorsement from the relevant state authorities.

**Table 1:** Management measures in the Sainte Luce lobster fishery.

Measure	Detail	*Dina*	*Lalana*
No take zone (NTZ)	13 km^2^ area periodically closed to lobster fishing (Fig 1). Openings of the NTZ were: 2014, August to September, inclusive; 2015, July to September inclusive; and 2016, April to May and August to September, inclusive.	X	
Minimum landing size (MLS)	20 cm	X	X
Landing restriction	Landing or sale of berried females	X	X
Gear restrictions	Snorkelling/diving equipment prohibited	X	
National closed season	1^st^ October to 31^st^ December inclusive		X

Management measures as dictated by *dina* (customary local law) and *lalana* (national legislation), in the Sainte Luce lobster fishery, Madagascar. An ‘X’ indicates the measure is specified by this form of legislation.

In March 2016, the Riaky Committee, reflecting wishes expressed by the community held a meeting of stakeholders during which it was agreed to hold two openings (April to May and August to September) in 2016 (Table 1). A combination of socio-economic and ecological reasons for this change were discussed, including: April-May was the period when the fewest females are berried; sea conditions are roughest August to September, thus an NTZ opening offers nearshore fishing in comparative safety in this period; and school fees have to be paid in October [32].

### Long-term trends

National annual lobster catch between 1976 and 2012 was obtained from the Food and Agriculture Organisation global production statistics [36]. Entries prior to 1976 were uniformly 100 tonnes and were discarded on the presumption that they were estimates of limited reliability. Annual catch between 1991 and 2015 at a regional and local (Sainte Luce) scale, maintained by Les Directions Régionales des Resources Halieutiques et de la Pêche (DRRHP, the regional fisheries authority), were obtained from URL. No time series for effort or CPUE were identified.

### Fishery monitoring

Fishery monitoring consisted of two survey types, a catch/effort survey and a catch composition survey. A participatory approach to data collection was adopted; data were used by the Riaky Committee and community to inform decision making, with technical assistance from the NGO partnership [32]. A member of the Sainte Luce community was employed to collect data, assisted by international volunteers, following training and initial supervision. For both survey types an opportunistic approach to sample selection was adopted to maximise sample sizes, minimise inconvenience to fishers and avoid catch degradation. Where the arrival of boats was suitably staggered or where volunteer assistant data collectors were available, it was possible to conduct both surveys described on all boats. On occasions where this was not feasible the catch/effort survey was prioritised.

Survey design was informed by coastal management monitoring guidelines for the Western Indian Ocean [37]. Prior to data collection, survey methodologies were subject to internal ethics review by both SEED Madagascar and ONG Azafady. Following this, permissions were sought from relevant Madagascan authorities: DRRHP, URL and Chef Fokotany Sainte Luce (elected mayor of the Sainte Luce community). The purpose of the research and methodologies were presented at a number of open community meetings in Sainte Luce. Each survey was subject to participants’ verbal consent. No individually identifiable data were collected in this study. Prior to weighing and measuring lobsters, permission was sought from fishers and *rabbateurs/collecteurs* at the point of sale. Ethics approval is not required for marine invertebrate research in Madagascar. In *lieu* of relevant country specific guidelines, lobster handling protocols were in accordance with the principles of the Australian Government National Health and Medical Research Council code of practice for the care and use of animals for scientific purposes [38]. Catch composition surveys were only conducted when there were sufficient personnel available to ensure lobster were handled quickly and efficiently. Catch composition surveys were performed *in situ* (on the beach) to minimise air exposure and temperature fluctuation, with immediate return to *rabbateurs/collecteurs* for processing.

### Catch/Effort survey

The catch/effort survey methodology was adapted from Stamatopoulos [39] and allows estimation of annual catch disaggregated by month (see Statistical approach). Sampling was conducted on approximately 15 days per month, February to September 2015 inclusive (n = 120 survey days). This represents a full season with the exception of January, which was used for methodological design and data collector training, with data excluded from analyses. Additionally, the catch/effort survey was performed at the Main landing site in 2016 on approximately 15 days per month (n = 84 survey days), from January to May inclusive, capturing the initial NTZ closure and subsequent first opening of the 2016 season (Table 1).

A survey consisted of visiting both landing sites (Main and Lodge) at dawn and estimating the total number of boats at sea by counting the drag marks left in the sand by launching boats. The number of inactive boats—seaworthy boats remaining on the beach—was also counted. The data collector(s) would then be stationed at one of the two landing sites until >80% of boats had returned. The number of survey days spent at each landing site per month varied to approximately represent the proportion of the fleet operating from each. This varied from month to month with the majority of boats operating from the Main beach in all months.

On days where one or more boats were active, boats were sampled on an opportunistic basis as they returned to the landing site, with the sample size always >50% of the number estimated to have gone to sea (from the count of drag marks). For each boat sampled that had been engaged in lobster fishing the following was determined: number of fishers, number of pots checked, number of lobster caught and total weight of the catch (± 10g, Brecknell digital scales).

### Catch composition survey

Catch composition surveys were undertaken alongside catch/effort surveys (n = 51 survey days), covering all of the months in the 2015 survey period. For a subsample of boats in the catch/effort survey, the entire catch was sampled. The following was determined for each lobster: species, total length (TL), carapace length (CL), sex and for females whether they were berried. The CL was measured (± 0.5 mm) from the base of the frontal horns to the posterior edge of the carapace. The TL was measured (± 0.5 mm) from the rostrum to the posterior edge of the telson, having dorso-ventrally flattened the individual. Sex was determined by the presence of either biramous pleopods (females) or uniramous pleopods (males). Berried status of females was determined by visual inspection for the presence of an egg mass.

### Statistical approach

Estimated total landings for 2015, disaggregated by month were calculated as follows. Monthly landings (*L*) were calculated per Equation 1, *L* = *E* × *C* where: *C* is the mean observed CPUE from sampling effort for that month and *E* is the effort. Effort (*E*) is calculated per Equation 2, *E* = *D* × *B* × *F* where: *D* is the number of days in the month, *B* is the Boat Activity Coefficient (BAC) and *F* is size of the fleet. The fleet size (*F*) was calculated for each month as the mean total boats (at sea and inactive) on days where both landing sites were surveyed with an additional five boats added to each monthly estimate. These additional boats represent those based upstream on the river (Fig 1), instead of the Main or the Lodge, but which sell their catch to *rabbateurs/collecteurs* at the Lodge landing site. This was assumed to be five boats based on data collectors’ observations and discussions with fishers.

The mean BAC was calculated for each month. This was achieved using all Main landing site surveys where fishers from sampled boats were asked if they had been engaged in lobster fishing. The observed sample ratio of boats lobster fishing to undertaking any other activity was then applied to the number of boats at sea and inactive boats counted at the start of the survey. The BAC for the fleet was calculated using only observations from the Main landing site. This was because of the small number of boats operating from the Lodge (typically <10). The inherent variability in activity at this landing site would have been unrepresentative when applied to the whole fleet. The BAC (*B*) for each Main landing site survey was calculated per Equation 3, *B* = *A* ÷ *T* where: *A* is the number of active boats (lobster fishing) and *T* is the total number of boats (at sea and inactive).

For the purpose of calculating monthly mean CPUE (*C*) and BAC (*B*), minimum sample sizes were determined *a priori* to exceed the 90% accuracy threshold as per Stamatopoulos [39, 40]. In all cases the sample sizes exceeded the minimum, with the exception of data from January, which were excluded from analyses. The mean of the estimated landings for February and March was used as an estimate for January in calculating the estimated total landings for 2015.

Data from catch/effort surveys at the Main landing site in 2015 and 2016 were used to model the relationship between CPUE and NTZ status. The CPUE (lobsters boatday^-1^) data were zero-inflated [41]. Approaches for modelling discrete zero-inflated data, specifically Zero-Inflated Poisson (ZIP), Zero-Inflated Negative Binomial (ZINB), Zero-Altered Poisson (ZAP) and Zero-Altered Negative Binomial (ZANB), are well described in the literature [41]. The source of zero-inflation was considered to be ‘true’ or ‘good’ zeros [41, 42], as the zeros represented boats landing no lobsters–a common occurrence–rather than an error in measurement. Whilst ‘bad’ or ‘false’ zeros could arise from non-reporting by fishers, or hiding undersized or berried lobsters, the use of a community data collector and the fisher/researcher relationship was such that this eventuality was assumed to be rare or non-occurring. Zero-altered models (ZAP and ZANB), also referred to as hurdle or two-part models, were selected as these do not assume zeroes are ‘false’ and best represent the underlying process in this context [41].

The analysis was performed in R version 3.0.2 [43], using the PSCL package version 1.4.9 to fit the models [44, 45]. Zero-altered models are partitioned into two parts. In the first part (zero hurdle model), a binomial model is used to model the probability that a zero value is observed. In the second part (the count model), the non-zero data is modelled using a truncated Poisson distribution, in the case of ZAP, or a truncated negative binomial distribution, in the case of ZANB [42]. For both parts, the response variable (CPUE) was modelled using the following explanatory variables and their interactions: NTZ status (categorical, 2 levels, open and closed) and number of pots (numeric). Akaike’s Information Criterion (AIC) and a likelihood ratio test (χ^2^) was used to compare the two full models (ZAP and ZANB), selecting the one that best fit the data. Stepwise model simplification was conducted using likelihood ratio tests (χ^2^) to produce the minimum adequate model (MAM). Model validation was undertaken as per Zuur *et al*. [42], by plotting residuals against fitted values.

A Kolmogorov-Smirnov test was used to compare the size class distributions of lobster from sampled catch to determine if these differed between the period of NTZ closure and opening.

## Results

### Long-term trends

**Fig 2:**
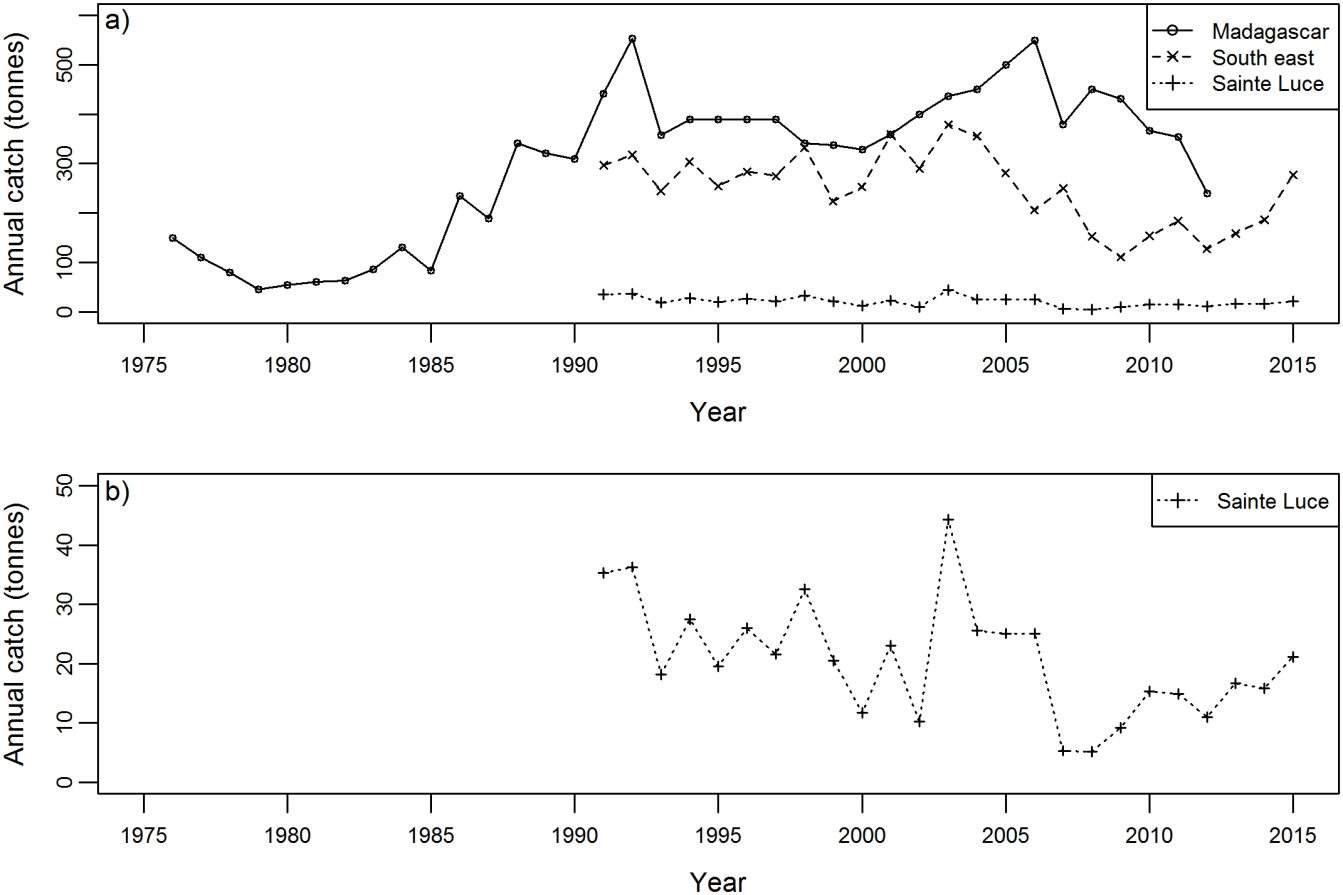
Long-term trends in annual lobster catch. Annual catch of lobster (tonnes) for the a) national (Madagascar), regional (south east) and local (Sainte Luce) scale and b) local (Sainte Luce) scale. Data: national, FIGIS (2015); regional and local, DRRHP/URL.

A time-series for annual catch of lobster at a national, regional and local scale (Sainte Luce) is shown (Fig 2).

### Catch/Effort data

In the 2015 season the mean number of boats in the fishery was 74.1 (sd = 13.3, n = 115), with a mean number of fishermen per boat of 3.6 (sd = 0.7, n = 2065) and a mean number of pots deployed per boat of 22.0 (sd = 13.5, n = 2062).

The ZANB provided the best fit of the CPUE data. The interaction between the NTZ status and the number of pots was significant in the zero hurdle model component (χ^2^ = 9.710, df = 1, p = 0.002) and in the count model component (χ^2^ = 32.129, df = 1, p < 0.001), thus the full model was the MAM.

**Fig 3:**
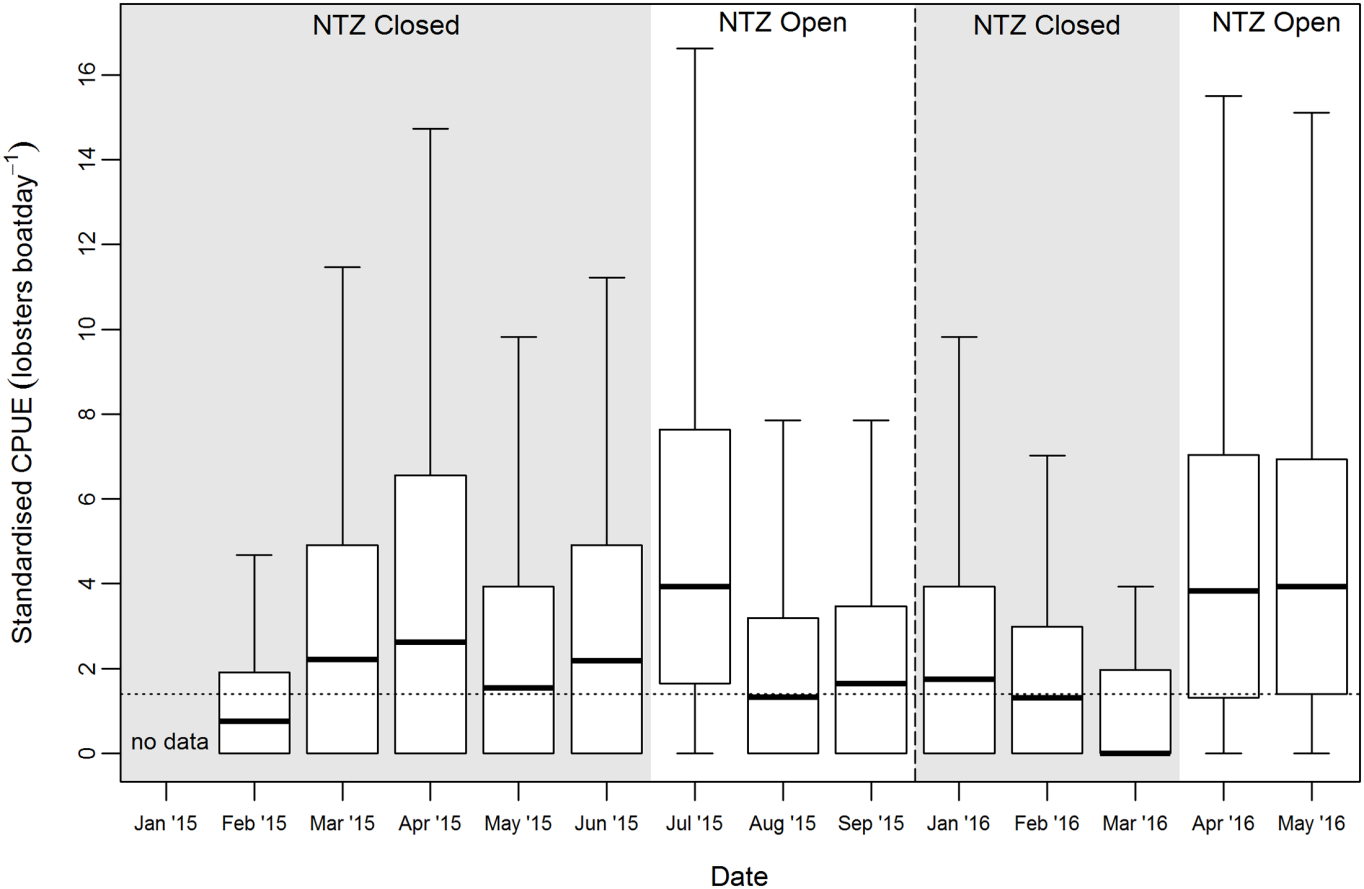
Variation in CPUE between months. Box-whisker plot showing standardised CPUE (lobsters boatday^-1^) in relation to the opening and closure of the NTZ. CPUE was sampled (n = 3,103) from the Main landing site, Sainte Luce, throughout the 2015 season (January to September) with the exception of January and from January to May 2016, inclusive. CPUE was standardised to the mean number of pots used per boat for the entire survey period, 19.6 pots (sd = 11.4, n = 3,103). Periods of NTZ closure (grey background) and opening (white background) are shown. The 2015 national closed season (October to December, inclusive) is indicated (dashed line). A reference line (dotted) shows the median CPUE for periods of NTZ closure, 1.40 lobsters boatday^-1^ (interquartile range = 0–3.27, n = 1,180).

The highest CPUE was observed during periods of NTZ opening (Fig 3). In 2015 differences in CPUE and effort resulted in variation in the estimated catch between months, with July (first month of NTZ opening in 2015) having the highest estimated catch of 4,593 kg (Table 2).

**Table 2:** Estimated lobster landings for 2015 from the Sainte Luce lobster fishery.

Month	Days	Survey occasions	Boat Activity Coefficient	Number of boats	Effort	CPUE	Estimated catch	Price	Estimated value
	*D*	*n*	*B*	*F*	*E = D*.*B*.*F*	*C*	*L = E*.*C*	*P*	*V = L*.*P*
					(boatsdays)	(kg boatday^-1^)	(kg)	(MGA kg^-1^)	(MGA)
January	31	-	-	-	-	-	1,015*	15,000	15,230,654
February	28	11	0.74	70	1,348	0.86	1,255	15,000	18,830,197
March	31	13	0.47	68	930	0.77	775	15,000	11,631,111
April	30	12	0.67	73	1,373	1.07	1,580	15,000	23,696, 567
May	31	17	0.19	91	495	1.02	536	15,000	8,039, 411
June	30	15	0.09	94	245	0.58	149	15,000	2,240,704
July	31	19	0.80	82	1,926	2.24	4,593	20,000	91,865, 265
August	31	17	0.80	79	1,827	0.78	1,531	20,000	30,629,189
September	30	16	0.50	70	965	0.53	554	20,000	11,081,059
2015 season							11,990	-	213,245,089

Estimated total landings for 2015 disaggregated by month for the Sainte Luce lobster fishery, Madagascar. Calculated from catch/effort survey occasions in 2015 (n = 120). Estimated value for each month was determined by multiplying the estimated catch by the price received at the point of first sale, when fishers sell catch to *rabbateurs/collecteurs*. The price changed between June and July, see text.

*No data was collected in January; the estimate is the mean of the estimated landings for February and March.

### Catch composition data

**Fig 4:**
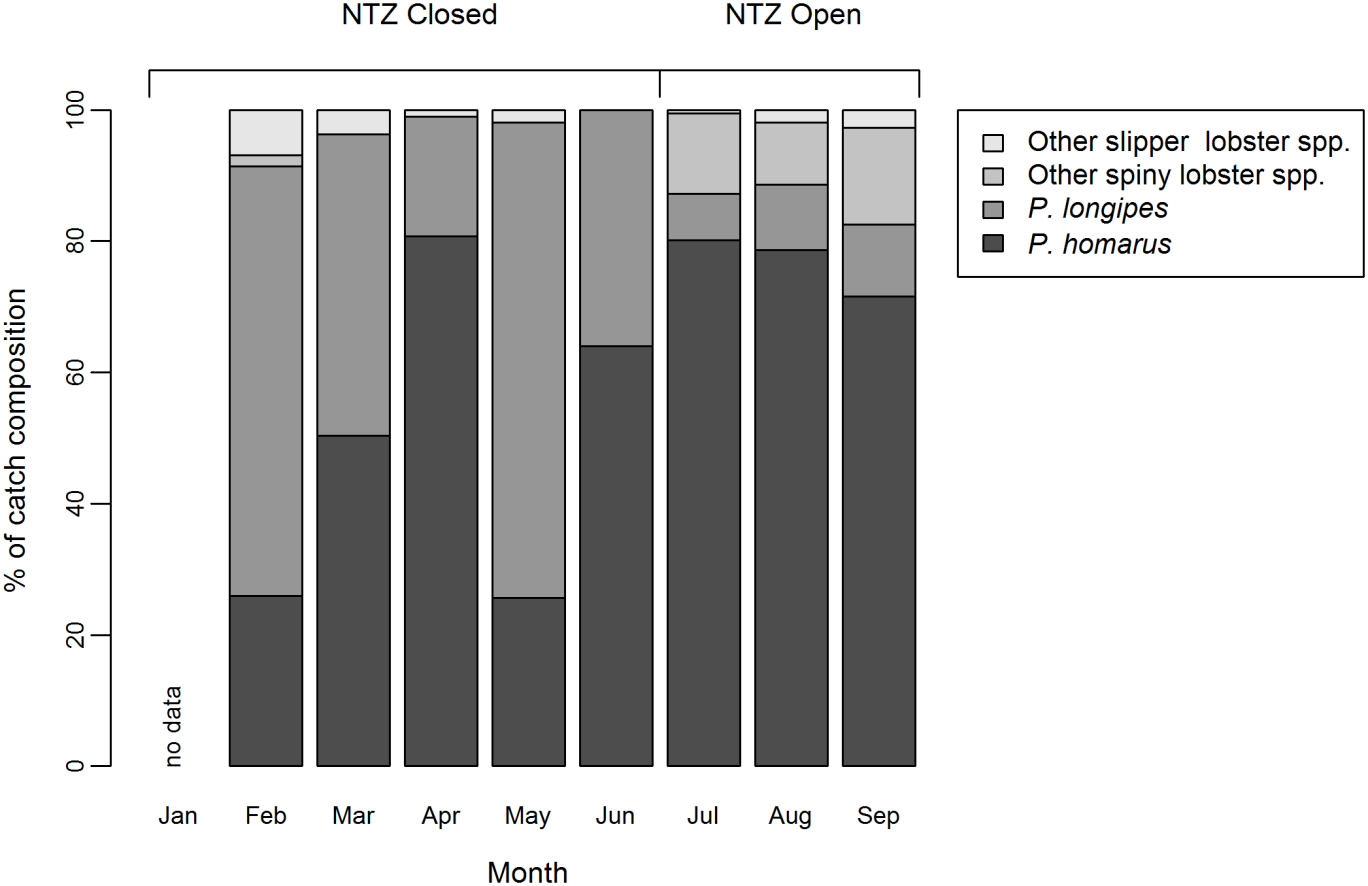
Variation in catch composition between months. Species catch composition by month (n = 1,919). Showing dominant species, *P*. *homarus* and *P*. *longipes* with other slipper lobster species (*S*. *squammosus*, *P*. *antarticus* and *A*. *regalis*) and other spiny lobster species (*P*. *ornatus* and *P*. *penicillatus*). The period of NTZ closure and opening is indicated. Catch sampled February to September 2015 inclusive, from the Sainte Luce lobster fishery, Madagascar.

**Fig 5:**
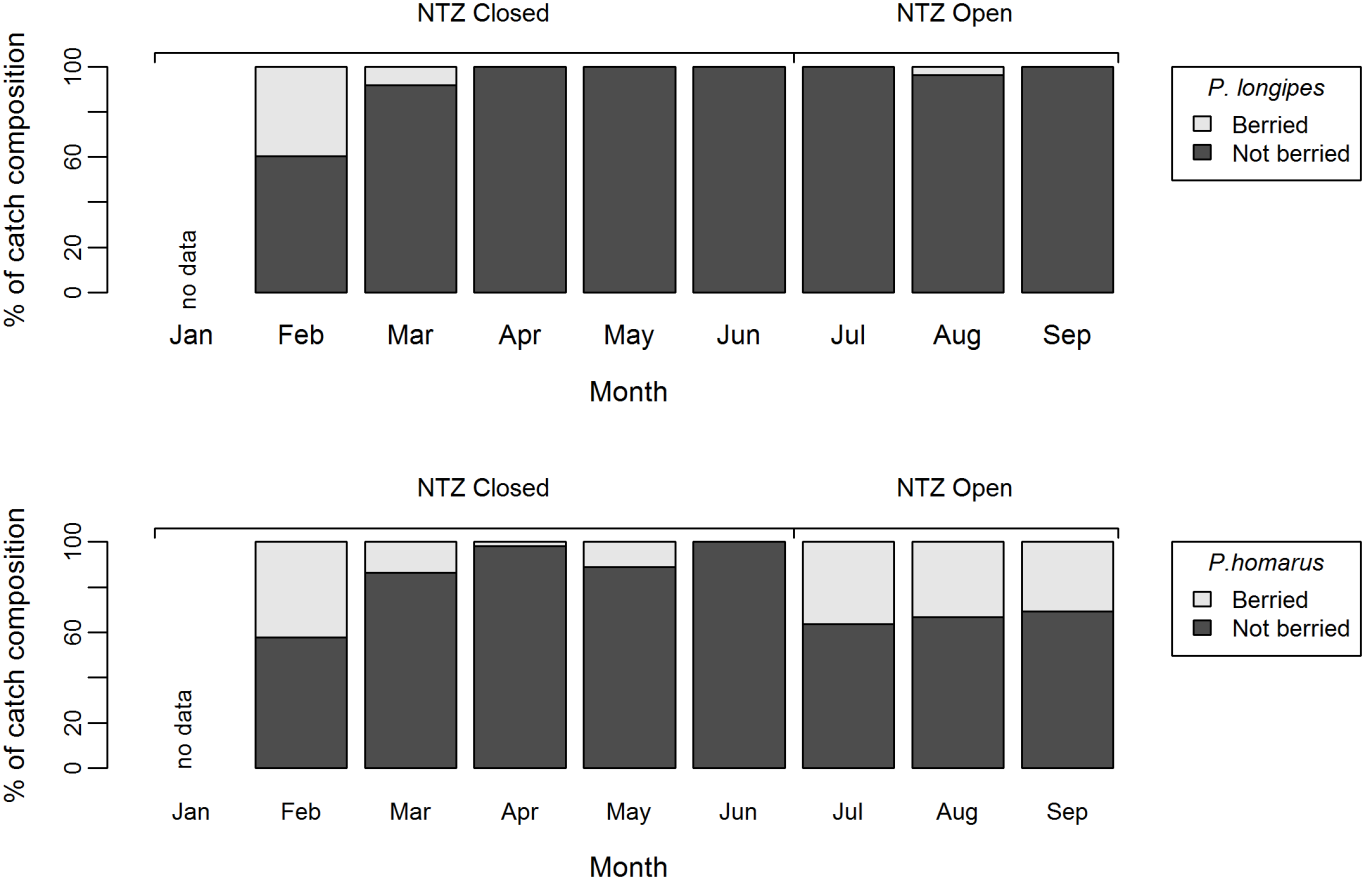
Variation in the proportion of berried females lobster between months. The proportion of female lobsters, *P*. *longipes* (n = 337) and *P*. *homarus* (n = 593), which were berried by month. The period of NTZ closure and opening is indicated. Catch sampled February to September 2015 inclusive, from the Sainte Luce lobster fishery, Madagascar.

A total of 7 lobster species were identified, composed of four spiny lobster species (*Panulirus homarus*, *P*. *longipes*, *P*. *ornatus*, *P*. *penicillatus*) and three slipper lobster species (*Scyllarides squammosus*, *Parribus antarticus* and *Arctides regalis*). The identification of *A*. *regalis* (only two individuals were encountered) is provisional, pending genetic analysis (P. Clark, Natural History Museum, pers. comm.). *A*. *regalis* has a wide Indo-West Pacific distribution but a comprehensive list of records in the literature [46] does not include Madagascar. The author was unable to identify any previous records for this species in the scientific or grey literature. Additionally, *P*. *versicolor* was observed by the author on one occasion, though not as part of a formal survey. Two of three *P*. *homarus* subspecies, *P*. *homarus homarus* and *P*. *homarus ruebellus* [47, 48], were identified in catch composition surveys, the ratio of which was not recorded. In 2015, *P*. *longipes* and *P*. *homarus* dominated the catch, with *P*. *longipes* more prevalent February to June, the period of NTZ closure, and *P*. *homarus* more prevalent July to September, the period of NTZ opening (Fig 4). The observed sex ratio of *P*. *homarus* and *P*. *longipes* in the sampled catch was close to 1:1 (*P*. *homarus*, 53.1% female) and (*P*. *longipes*, 52.4% female). The proportion of females (*P*. *homarus* and *P*. *longipes*) which were berried is provided in Fig 5.

**Fig 6:**
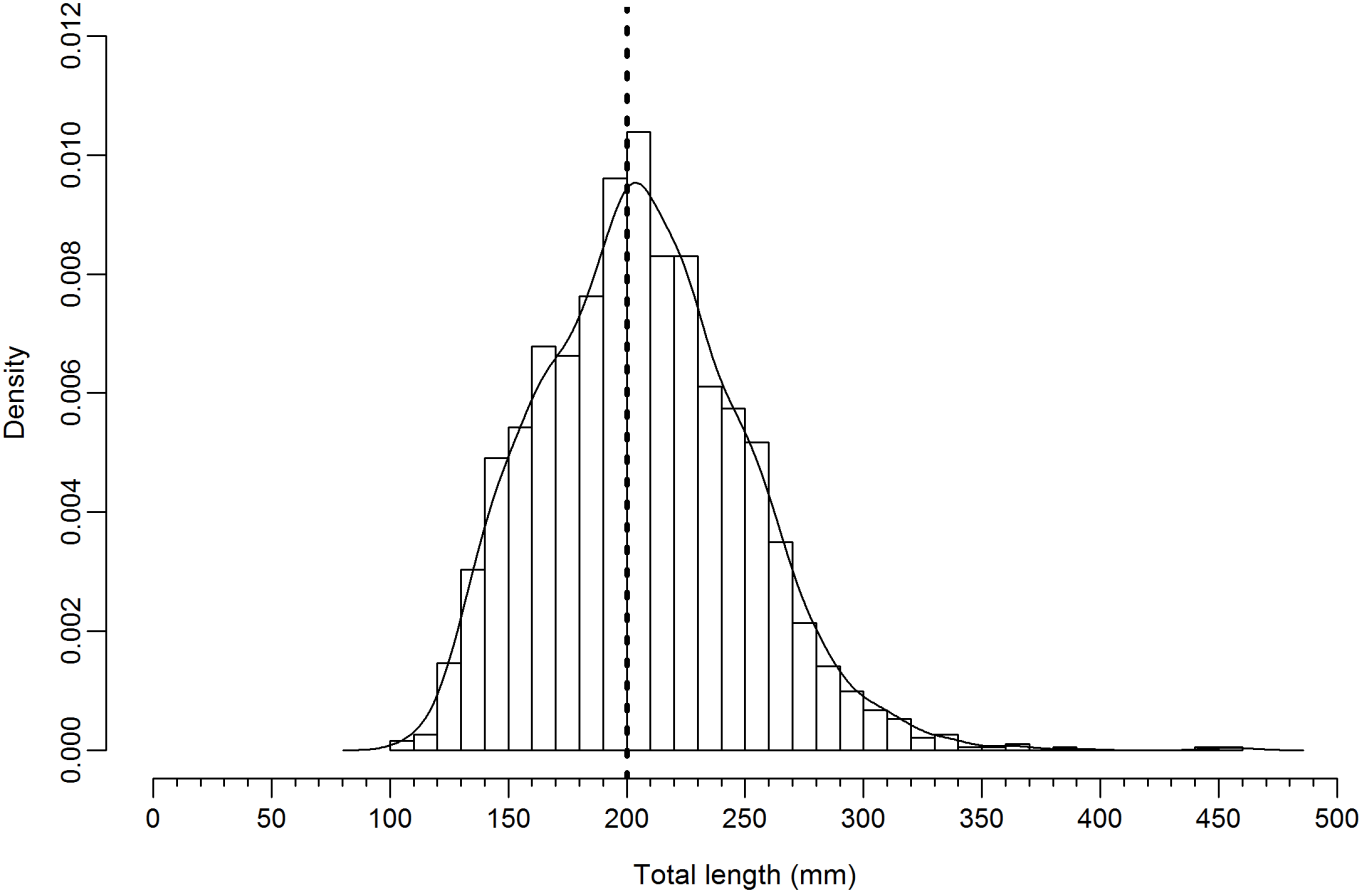
Size classes of lobster. Histogram of total length of lobsters of all species sampled from catch, February to September 2015 inclusive (n = 1,915), caught in the Sainte Luce Lobster fishery, Madagascar. The MLS (200 mm) is shown (thick dashed black line), with kernel density estimate (bandwidth = 8.565) drawn (solid black line), bin widths are 10 mm.

**Fig 7:**
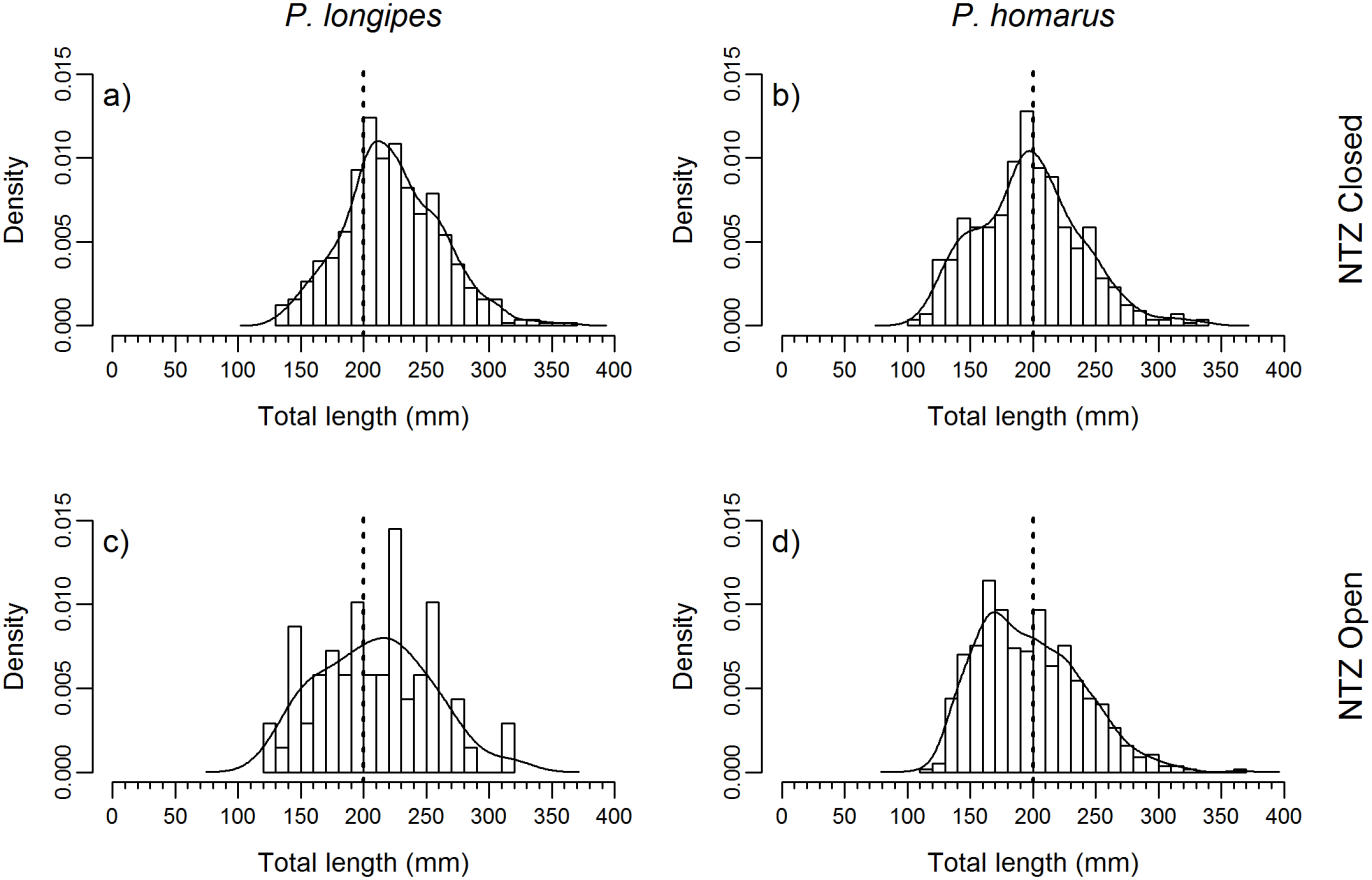
Size classes of lobster caught during closure and opening of the NTZ. Histogram of total length (mm), with 10 mm bin widths, of lobsters sampled from the Sainte Luce lobster fishery, Madagascar. Sampling from two periods: when the NTZ was closed (February to June inclusive), and when the NTZ was open (July to September inclusive). Histograms with kernel density estimates overlaid are shown for a) *P*. *longipes* NTZ closed (bandwidth = 9.43, n = 572), b) *P*. *homarus* NTZ closed (bandwidth = 10.53, n = 563), c) *P*. *longipes* NTZ open (bandwidth = 17.12, n = 69) and d) *P*. *homarus* NTZ open (bandwidth = 10.32, n = 569). The MLS (200 mm) is drawn (black dashed line) for reference.

The size structure of the catch for all species is presented, with 42.8% of lobsters sampled <MLS (Fig 6). The size distribution of lobsters (*P*. *longipes* and *P*. *homarus*) in relation to the NTZ (closed and open periods) is provided in Fig 7. The size distribution of *P*. *longipes* caught during NTZ closure was significantly different compared with that during NTZ opening period (Kolmogorov-Smirnov test, D = 0.197, p = 0.017); in contrast, the *P*. *homarus* size distribution was not significantly different between these two periods (Kolmogorov-Smirnov test, D = 0.073, p = 0.096).

For the purpose of comparison with the wider literature, the relationship between TL and CL is shown, disaggregated by sex, for all species where the sample size at level of sex was sufficiently large (S1 Fig).

## Discussion

### Long-term trends

Lobster catches at a national, regional and local level are below historic peaks, in agreement with the perceptions of local fishers, the regional industry and authorities [27, 32]. A study of the regional fishery concluded that it was highly likely that the fishery would collapse within ten years [29]. Elements of the study were flawed, drawing one of its conclusions based on an extrapolation from just four CPUE data points: the predicted collapse has demonstrably not occurred. Nevertheless, the data presented by Sabatini *et al*. [29] do support the conclusion that there has been a long-term decline in catch. It is unlikely that this can be attributed to decreased fishing effort. Madagascar has a high population growth rate of 2.8% per year [15], which is thought to be higher in coastal regions [16]. This has probably resulted in a greater number of lobster fishers given that extreme poverty is most pronounced in the southeast of Madagascar [15], lobsters are a comparatively high value commodity, and there are few barriers to entering the fishery.

Historically *P*. *homarus* has accounted for the majority of the regional catch [27, 29]. Catch composition in this study (*P*. *homarus*, 58.8%*; P*. *longipes* 33.3%) was markedly different from that previously reported (*P*. *homarus*, 93.09%*; P*. *longipes* 6.36%) for the northern area of the regional fishery in which Sainte Luce is situated [27]. Depth preference reduces interspecific competition between *P*. *homarus* and *P*. *longpipes*, with the former favouring shallow inshore reef and the latter found at greater depths [47, 49, 50]. Increasing contribution of *P*. *longipes* to catch composition may indicate fishers are having to travel further offshore and exploit deeper reefs to compensate for declining catches, as has been observed in numerous other SSFs [51, 52]. An analysis of catch composition at a regional level would confirm this hypothesis.

Increases and changes in the spatial distribution of effort may have maintained catches (or slowed the rate of decline), masking prolonged and significant declines in regional stock of lobster. The Mozambique channel represents a barrier to larval dispersal of *P*. *homarus ruebellus* [53], suggesting that the southern Madagascan population of at least this subspecies should be regarded as a separate management unit to exploited populations along the east African coast. In light of the apparent declines identified, there is a need for effective precautionary management at a regional level, potentially through the adoption of community-based management.

### Impact of the NTZ on landings

The CPUE was significantly different depending on the status (open or closed) of the NTZ. In July 2015, when the NTZ was opened after a six month closure, the CPUE was the highest estimated for that season, either as the number of lobsters boatday^-1^ (Fig 3) or weight (kg) lobster boatday^-1^ (Table 2). Accordingly, the greatest estimated catch for any month in the 2015 season was 4,593 kg in July (Table 2), a 435% increase in the mean CPUE from the preceding 5 months. This can be partly accounted for by a 260% increase in CPUE on the mean for the preceding 5 months. To some extent this was a self-fulfilling prophecy; fishermen expected higher catches and thus expended a greater level of effort, with July seeing a 118% increase in the number of boatdays. The effect was short lived with effort, CPUE and landings returning to typical levels in August and September (Fig 3 and Table 2). Intense fishing effort in response to NTZ openings has previously been reported elsewhere. For example in the Solomon Islands, effort within open NTZs was four to 60 times higher compared with continuously open reefs [54]. In addition in Fiji, openings have been associated with intense fishing effort [55]. Finally in Madagascar, there was a median 477.8% increase in effort, comparing 30 days before and after octopus NTZ openings at 36 sites [56].

In the absence of data from other fisheries in the region or suitable control sites, the increased CPUE in July 2015 and in April and May 2016 cannot be conclusively ascribed to the impact of the NTZ. However, there are no apparent ecological processes that would account for an increase in CPUE in July of one year followed by a similar effect in April and May of the subsequent year. In 2014, the first year of implementation, the NTZ was opened 1^st^ August. Whilst no data are available, the perception among stakeholders was that catch was significantly higher on opening in August 2014 [32]. This further suggests that increased CPUE is attributable to the NTZ opening, not an unrelated ecological phenomenon whose timing happened to coincide. It should be acknowledged that whilst the effect of the number of pots used on CPUE (lobsters boatday^-1^) was accounted for, there are other variables which may be important. This could in include the area fished as productivity likely varies between reefs and water temperature, which is known to effect lobster activity and catchability [57].

This raises the question of whether the increased CPUE is the result of increased lobster biomass in the NTZ. The nine and six month closure periods preceding opening in 2015 and 2016, respectively, are relatively short compared with the lifecycles of the target lobster species. The larval stages are adapted to prolonged life in the open ocean and wide dispersal [58]. Laboratory reared *P*. *homarus* larvae took 60 days to reach the sixth [59] of nine or ten phyllosomal stages [58]. The total duration of the larval stage likely results in considerable dispersal, especially as late stage larvae are seldom found within the shelf edge [58]. Therefore, local recruitment to the fishery of the settlement stage larvae (known as puerelus, CL 6-12mm) [60] seems unlikely. Furthermore, these are only the second and third years of NTZ operation. Thus it is doubtful that the closure has resulted in an increased population within the NTZ. The increased CPUE was observed in the number of lobster. Catch was dominated by *P*. *homarus* (Fig 4), with no significant difference in the size distribution seen (Fig 7). This is in accordance with the available knowledge on the growth rate of *P*. *homarus*. In the wild the interval between settlement and attaining a CL of 60 mm is 1–3 years [60], with growth rates slowing as lobsters get larger [61]. A male *P*. *homarus* with a CL of 60 mm has a total length of ~186 mm (S1 Fig) and so has not yet attained the fishery’s 200 mm MLS.

A more likely explanation for the increased catch is that this is simply the result of the concentration of effort (in time and space) in a productive area of the fishery, with CPUE and catch diminishing as the stock became locally depleted. Whilst not intentional, the decision to change the NTZ operation from one opening (July to September) in 2015 to two (April to May and August to September), provided a natural experiment, which supports this explanation as the same effect was observed after both openings. If this is the case, the observed higher CPUE is no different to the large catches associated with opening days of the season in many fisheries around the world.

### Ecological value of the NTZ

The communities on the west coast of Madagascar have been implementing NTZs for octopus since 2004 [22]. Discussing the transferability of this model, Oliver *et al*. [56] note that octopus with fast growth and short lifespans are ideal targets for these types of periodic closures and that the successful application of this model elsewhere would depend on both the bio-economic and governance context. Whilst there are some similarities between the octopus fisheries of the west coast and the lobster fishery in Sainte Luce (low size selectivity, low harvesting costs, widespread community participation, open access), crucial differences are that lobsters are comparatively slower growing and have longer lifespans.

However, a short term closure could provide a number of potential ecologically benefits to a lobster fishery. Lobsters with tropical and subtropical distributions are known to have year-round or extended spawning periods of over 6 months with pauses for moulting [62, 63]. The peak breeding season is thought to be October through to December, which was the rationale for a change in the law in 2004 moving the national closed season to this period [27, 29]. The data presented (Fig 5) do not contradict this, but also demonstrate that significant numbers of females of both *P*. *longipes* and *P*. *homarus* are berried in February and March. There are also significant numbers of *P*. *homarus* berried in July, August and September. In the context of low compliance with the ban on landing berried females (see below), the NTZ can offer protection to these spawners, in an area of key habitat with a significant lobster population. Closures synchronised with breeding periods are a well-utilised tool in lobster fishery management, reducing exploitation but also preventing disturbance to the stock at a critical phase of the life cycle [62]. Research on *P*. *interruptus* has demonstrated breeding seasons can vary between zones of a regional fishery, leading Vega [64] to propose a zonal closed season tied to the local timing of breeding. The NTZ model here could allow community managers to utilise local ecological knowledge to maximise benefits by timing spatial closures to local phenology. The Sainte Luce NTZ offers partial protection, with likely beneficial impacts on recruitment to the regional stock. This effect is skewed in favour of *P*. *homarus* as this species dominates in the NTZ (Fig 4) due to a habitat preference. The NTZ likely partially protects shallow inshore nursery grounds. This could be optimised by minimising fishing in areas which yield the highest number of immature and undersized lobsters, as was recommended for a small-scale lobster fishery in the Turks and Caicos Islands [65].

In addition to extended protection during a portion of the breeding season, the short opening period (2015, three months; 2016, two openings of two months) also increases the likelihood that individuals in the NTZ will not be caught in any given year. In the medium term this has the potential to result in lobster attaining larger sizes, shifting the size class distribution in favour of larger lobsters over several years. This may not necessarily be reflected in the NTZ, as large *P*. *homarus* have been shown to migrate to deeper reefs [66]. An increase in the number of large individuals in the population has the potential to positively increase recruitment, as fecundity of spiny lobsters is known to increase with size [62].

Whilst there is a growing body of knowledge relating to permanent no take reserves and MPAs, there has been little empirical evidence in the literature on the efficacy of short-term closures, or periodic NTZs, on biodiversity and biomass [67, 68]. Reviewing periodic closures in SSFs, Cohen and Foale [69] identified benefits in fisheries for short-lived, fast growing taxa or where fishing pressure is low, but not where pressure is intense or target species long-lived. Similarly, modelling of short opening and closing cycles has shown it can result in increased biomass and yields for short-lived, fast growing, sedentary species [70]. In terms of lobsters there remains a lack of knowledge and consensus even for the impacts of marine reserves and MPAs [71], which likely varies between species and biophysical context. No examples of NTZs specifically designed for lobsters in SSFs could be found in the literature. Gendron and Brêthes [57] used a spatially explicit model to explore the impacts on fishing mortality of different spatio-temporal allocations of fishing effort for *Homarus americanus*, including within season brief NTZ closures (3 weeks). The model showed closures to be successful in reducing exploitation rates. However, it is difficult to apply those findings in this context. The model relied on a good understanding of the inshore/offshore migration dynamics and seasonal variability in catchability. These are not known here. Further, the model does not account for concentrated effort in previously closed areas, as has been shown to occur in this fishery.

It remains to be seen whether the potential ecological benefits as outlined above, or indeed others, will be realised. Over the past decade the use of NTZs for octopus has been replicated over 100 times along some 350 km of the west coast of Madagascar [22]. Whilst short-term increases in CPUE and positive economic impacts associated with these closures have been reported [56, 72], no comprehensive assessment has been made of long-term population effects on the target species or ecosystem impacts. This would be useful in assessing the NTZ model given its proliferation both in Madagascar and further afield.

### Economic value of the NTZ

Ecological considerations aside, fishers earned significantly more money during the July 2015 opening as a result of the NTZ. There was a 613% increase in the estimated value of the July catch compared with the mean of the estimates for the preceding 5 months. This was a product of two factors: increased catch and an increase in the price fishers received. There was considerable anticipation among the community and wider industry in the months preceding the opening in 2015 (pers. observation). The *opérateurs* were keen to maximise their market share and exclude competitors. A meeting held between all stakeholders in June 2015 was instrumental in increasing the price from 15,000 MGA (~$4.50 USD) kg^-1^ to 20,000 MGA (~$6.00 USD) kg^-1^ from July onwards [32, 73]. Although the increase in CPUE was not sustained until the end of the season the increased price was, subsequently reverting to 15,000 MGA kg^-1^ in January 2016. The NTZ opening in April 2016 again saw price increases to between 20,000 and 25,000 MGA ($6.00 and $7.50 USD) kg^-1^, driven by the same mechanism.

As observed in other SSFs in the Caribbean [24], the processors/exporters in the regional lobster fishery hold considerable influence over other actors in the value chain. In Sainte Luce the majority of fishers sell lobsters to two *opérateurs*, Madapêche and Martin Pêcheur [31]. These companies are the founding and controlling members of *Groupement des Opérateurs Langoustiers Du Sud* (GOLDS), an entity representing the interests of lobster exporters in the south. Only a small proportion of boats in the fleet (<10) are owned by fishers in Sainte Luce, with the majority owned by *opérateurs*. This constrains fishers to sell to particular *collecteurs*, working for the *opérateur* that supplied the boat. Thus prices are depressed, as it prevents fishers from seeking the most competitive price. Those with their own boats are able to achieve prices that are around 2,000–5,000 MGA kg^-1^ ($0.60–1.50 USD) higher [32]. In this context, a management measure which brings about a 33% increase in price at the bottom of the value chain may be of considerable value. Monnereau and McConney [24] argue that spreading the benefits and power more evenly along the value chain and diminishing the role of powerful market entities, is a key step in enhancing the governability of SSFs.

### Compliance with management measures

In agreement with previous reports [27], compliance with the MLS and prohibition on landing berried females was low (Figs 5 and 6), with 48.2% of the catch <MLS. Consideration of typical earnings offers insights into one underlying driver of non-compliance. The median catch was 0.18 kg lobster fisher^-1^ day^-1^ (interquartile range = 0–0.44, n = 2100), which equates to 2,700 MGA (~$0.81 USD) fisher^-1^ day^-1^ from the primary livelihood activity for most households. Levels of non-compliance are thus unsurprisingly high, in a context where *rabbateurs*/*collecteurs* are willing to purchase illegal (undersized and/or berried) catch. This highlights that fishers simply cannot afford to adopt sustainable practices, emphasising that changes in the value chain are a necessary pre-condition for it to be economically feasible for fishers to adopt sustainable practices.

In lobster fisheries, the protection of reproductive capacity has often been a secondary consideration to acceptable market size in setting MLSs [62]. The current MLS of 20 cm, established in 1962, is unlikely to be optimal given it applies to eight different species. A further consideration is that size at maturity (SAM) is known to vary spatially (*P*. *homarus*: [74]) and with exploitation pressure [75]. However, enforcement of an inappropriate MLS may be both economically detrimental and of limited benefit in protecting the regional stock. Approaches for determining the SAM in *P*. *homarus* [76, 77], could be used for the dominant species in this fishery to inform the revision of the current MLS. To facilitate comparison with the wider literature and other management regimes, which tend to use CL rather than TL, the CL-TL relationship is presented (S1 Fig).

Although the MLS and prohibition on landing berried females are included in the *dina*, this was to ensure that it could pass judicial review rather than by community demand (E. Ellis, ONG Azafady, pers. comm). In contrast, the NTZ was designed at the community level, demonstrated by their decision to alter the opening periods in 2016. The data presented here do not directly assess the level of compliance among fishers with the NTZ. However, it is reasonable to infer that the effect observed during NTZ opening would not have occurred without widespread compliance with the closure. In a context where NGOs play a strong role in designing management plans and zoning natural resources [78], it is an important finding that it is only the community-led measures that appear to be successfully enforced.

### NTZ as a catalyst for community-based fishery management

The term ‘community catalyst hypothesis’ was used by Oliver *et al*. [56] to describe the effects of the short-term impacts of NTZ openings (temporary increases in daily catch and earnings), on community attitudes to fishery management. Specifically, that this may serve to increase community buy-in to fishery management, lead to the introduction of further management measures and adoption of community-based fishery management in other fisheries. The Sainte Luce NTZ was established in 2014 and appears to have impacted attitudes in the adjacent fishing communities of Esohihy [73] to the north and Itapera to the south. In 2015, Esohihy implemented a NTZ (closed 1^st^ October to 31^st^ March inclusive) and established a committee of fishery managers from the community without external support from an NGO. In 2016, Itapera initiated a NTZ (closure period under discussion) and set up a management committee [32]. These communities are now formally included in the on-going project implemented by the NGO partnership. Of the approximately 40 communities spread along ~450km of coastline [29], only those immediately adjacent have adopted NTZs. This, combined with the timing (established within 2 years), makes it reasonable to infer that this can be attributed to the catalytic effect of the Sainte Luce NTZ. It has not yet led to the introduction of other management initiatives or fostered compliance with other existing measures. Focusing on a single initiative to develop community buy-in for fishery management followed by stepwise introduction of further measures designed by the community may be the most effective approach. It has been shown that compliance is lower in cases where numerous measures are simultaneously adopted [79]. The utility of NTZs for lobster within the regional fishery therefore depends on whether they prove to have long-term ecological benefits and/or bring about the adoption of, and compliance with, an effective suite of management measures at the community level.

## Conclusion

Madagascar’s lack of political stability, adequate funds or institutional capacity mean bottom-up approaches may offer the most viable solution for management of critically important marine resources [13]. In the context of a fishery experiencing catch declines, most likely due to over-exploitation, this study identified an example of community based-fishery management, using NTZs with significant increase in CPUE on opening. Whilst the short duration of NTZ closures employed in Sainte Luce may not accrue tangible ecological benefits in the short term, there have been important impacts on the value chain. This appears to be responsible for catalysing the adoption of community-led fishery management by adjacent fisheries. However, so far this has not increased compliance with, or support for, other management measures. Furthermore, it has previously been acknowledged that LMMA measures designed to produce short-term increases in catch may not necessarily enhance the long-term sustainability of fisheries [11]. Thus, NTZs are shown to be a useful tool but not a complete solution, not least because effective management of a regional stock cannot be achieved by communities acting in isolation. Successful implementation of bottom-up management, in the Fort Dauphin regional lobster fishery, as with other SSFs in Madagascar, will require regional replication of a suite of measures and support from the state and industry.

## Supporting information

S1 Text Alternative language abstract, French. (DOCX)

S1 Fig The relationship between total length and carapace length for four lobster species. The relationship between total length (mm) and carapace length (mm) for males (dark grey open circles, solid black line) and females (light grey crosses, dashed black line) for four species of lobster. With: a) *P*. *homarus* (n = 1099); b) *P*. *longipes* (n = 555); c) *P*. *penicillatus* (n = 88) and d) *S*. *squammosus* (n = 46). Catch sampled between February and September 2015 inclusive, from the Sainte Luce lobster fishery, Madagascar.
(TIFF)
