# Supplementary material for: Short-term impacts and value of a periodic no take zone (NTZ) in a community-managed small-scale lobster fishery, Madagascar
Source: PLoS One. 2017 May 18;12(5):e0177858. doi: 10.1371/journal.pone.0177858 (PMC5436820; doi:10.1371/journal.pone.0177858)
Supplement: S1 Text — (DOCX) [file pone.0177858.s001.docx]

# **S1 Text: Alternative language abstract, French**

La pêche aux langoustes à petite échelle de la côte sud-est de Madagascar représente la majorité des captures nationales et l'exportation, contribuant ainsi significativement à l'économie régionale. Les peu de données disponibles, montrent une diminution des captures, vraisemblablement due à une surexploitation. En réponse, la communauté de Sainte Luce a mis en place une aire marine protégée à gestion locale pour gérer leur pêche aux langoustes, comprenant une surface de 13 km^2^ périodiquement qualifiée une zone interdite de pêche (ZIP), fermée neuf mois par an. Les données de surveillance participative de la saison 2015 sont utilisées pour informer et évaluer la gestion.Le respect des mesures conçues et mises en place par consensus communautaire (ZIP et restrictions de matériaux de pêche) est élevé. En revanche, quant aux mesures dictées par la législation nationale (taille minimale de capture (TMC), période de fermeture nationale, interdiction de capturer des femelles grainées), ce respect est faible. Lors de l’ouverture de la réserve en Juillet 2015, une augmentation des prises estimée à 435% a été observée par rapport à la moyenne des cinq mois précédents. Cela a été le résultat de l'augmentation de l'effort et de la variation significative de la capture par unité d'effort (CPUE) entre les mois ; le moi de Juillet présentant la plus haute CPUE. L'effet sur la CPUE est de courte durée, d'une durée inférieure à un mois. Dans la deuxième année de fonctionnement du ZIP, il est peu probable que des avantages écologiques tangibles aient été accumulés dans une telle courte période (neuf mois de fermeture), néanmoins la ZIP a eu des effets socio-économiques importants. Plus précisément, il a contribué à provoquer une augmentation de 33% du prix reçus par les pêcheurs, un effet significatif à la base de la chaîne de valeur. Ces augmentations temporaires de capture et de revenus apportées par l’installation du ZIP ont agi comme un catalyseur, encourageant la participation des communautés voisines à la gestion de la pêcherie, résultant à la naissance de deux ZIPs supplémentaires. Il reste à voir si cet effet catalytique conduira à la mise en place d'autres mesures de gestion ou accroîtra le respect de ceux qui sont n’ont pas observé ni mis en vigueur ces mesures. L'attention est attirée sur le fait que la législation nationale actuelle peut être sous-optimale et devrait être réexaminée. Des limitations de la gestion ascendante ont été identifiées. Une gestion réussie de la pêche régionale exigera que l'état et les industries soutiennent les communautés dans l'adoption de la gestion communautaire. La mesure de réserve temporaire ZIP ici considérée peut être un outil efficace pour atteindre cet objectif.

**Translation provided by: José Victor, Institut Halieutique et des Sciences Marines (ISHM)**
