# Supplementary figures and images for: Short-term impacts and value of a periodic no take zone (NTZ) in a community-managed small-scale lobster fishery, Madagascar
Source: PLoS One. 2017 May 18;12(5):e0177858. doi: 10.1371/journal.pone.0177858 (PMC5436820; doi:10.1371/journal.pone.0177858)

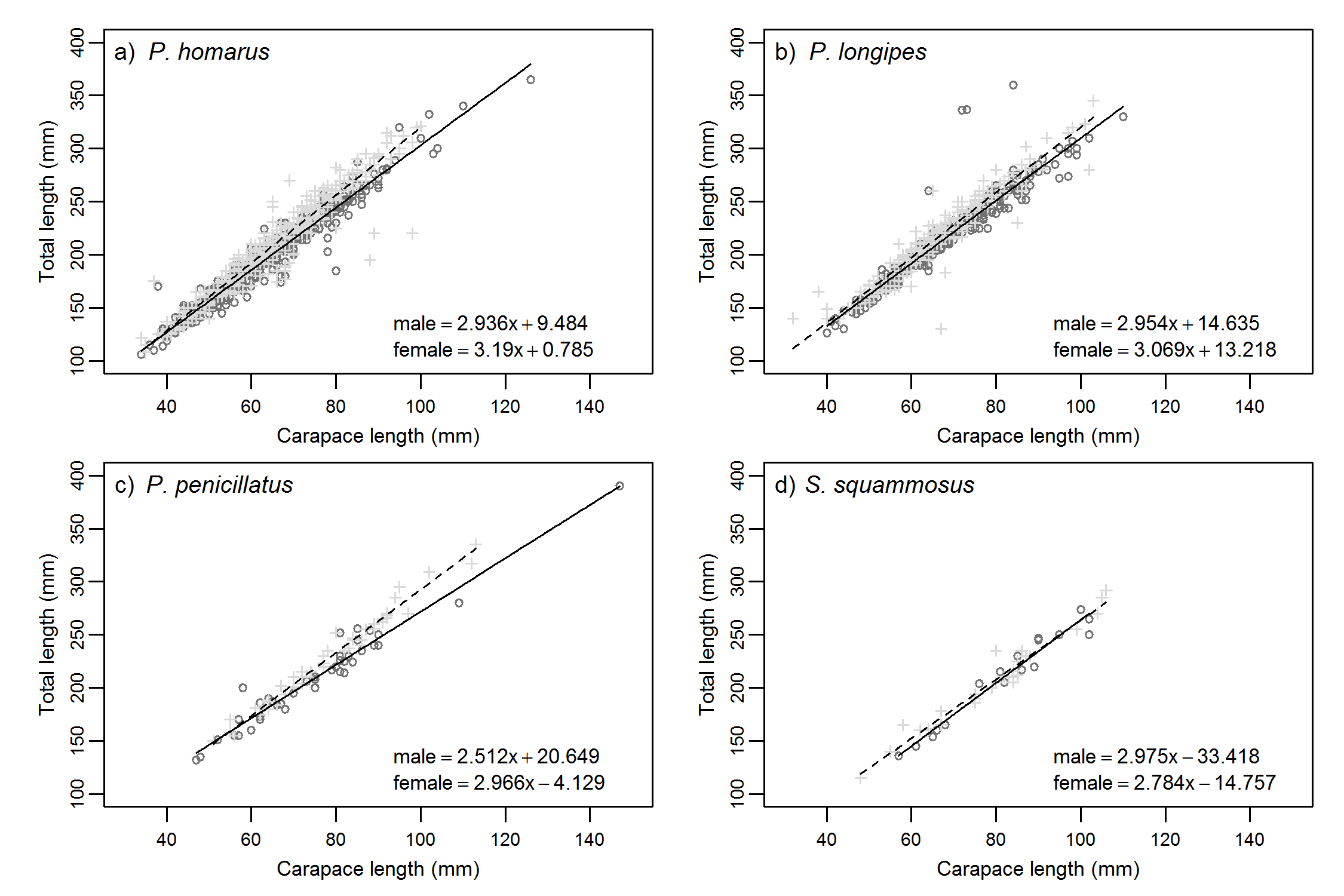

Supplement: S1 Fig — The relationship between total length (mm) and carapace length (mm) for males (dark grey open circles, solid black line) and females (light grey crosses, dashed black line) for four species of lobster. With: a) P. homarus (n = 1099); b) P. longipes (n = 555); c) P. penicillatus (n = 88) and d) S. squammosus (n = 46). Catch sampled between February and September 2015 inclusive, from the Sainte Luce lobster fishery, Madagascar. (TIFF) [file pone.0177858.s002.tiff]
